# Giving meaning to the scores of the Amsterdam instrumental activities of daily living questionnaire: a qualitative study

**DOI:** 10.1186/s12955-022-01958-2

**Published:** 2022-03-24

**Authors:** Mark A. Dubbelman, Caroline B. Terwee, Merike Verrijp, Leonie N. C. Visser, Philip Scheltens, Sietske A. M. Sikkes

**Affiliations:** 1 Department of Neurology, Amsterdam Neuroscience, Alzheimer Center Amsterdam, Vrije Universiteit Amsterdam, Amsterdam UMC, Location VUmc, P.O. Box 7057, 1007 MB Amsterdam, The Netherlands; 2 Department of Epidemiology and Data Science, Amsterdam UMC, Amsterdam, The Netherlands; 3 Division of Clinical Geriatrics, Department of Neurobiology, Care Sciences and Society, Center for Alzheimer Research, Karolinska Institutet, Stockholm, Sweden; 4 Faculty of Behavioural and Movement Sciences, Clinical Developmental Psychology and Clinical Neuropsychology, Vrije Universiteit Amsterdam, Amsterdam, The Netherlands

**Keywords:** Functional impairment, Clinical meaningfulness, Thresholds, Alzheimer’s disease, Dementia, Bookmarking

## Abstract

**Background:**

Everyday functioning is a clinically relevant concept in dementia, yet little is known about the clinical meaningfulness of scores on functional outcome measures. We aimed to establish clinically meaningful scoring categories for the Amsterdam Instrumental Activities of Daily Living Questionnaire (A-IADL-Q), representing no, mild, moderate and severe problems in daily functioning.

**Methods:**

Informal caregivers (*n* = 6) of memory-clinic patients and clinicians (*n* = 13), including neurologists and nurse specialists, working at various memory clinics in The Netherlands. In focus groups, participants individually ranked nine summaries of fictional patients from least to most impairment in daily functioning. Then, they placed bookmarks to demarcate the thresholds for mild, moderate and severe problems. Individual bookmark placements were then discussed to reach consensus. Clinicians completed a survey in which they placed bookmarks, individually.

**Results:**

While individual categorizations varied somewhat, caregivers and clinicians generally agreed on the thresholds, particularly about the distinction between ‘no’ and ‘mild’ problems. Score categories were no problems (*T*-score ≥ 60), mild problems (*T*-score 50–59), moderate problems (*T*-score 40–49), and severe problems in daily functioning (*T*-score < 40), on a scale ranging 20–80.

**Conclusions:**

Our findings provide categories for determining the level of functional impairment, which can facilitate interpretation of A-IADL-Q scores. These categories can subsequently be used by clinicians to improve communication with patients and caregivers.

**Supplementary Information:**

The online version contains supplementary material available at 10.1186/s12955-022-01958-2.

## Background

Impairment in daily functioning due to cognitive decline is a core characteristic of dementia [1]. Recent studies have shown that changes in daily functioning, in particular in ‘instrumental activities of daily living’ (IADL) [2], may occur well before dementia and even as early as the preclinical stage of Alzheimer’s disease [3–6]. IADL comprise cognitively complex activities such as doing grocery shopping, cooking, and using a computer, and as such, reflect cognitive functions in everyday life. IADL assessments can be helpful for monitoring disease progression and evaluating treatment effects [7, 8].

Impairment in IADL is fundamentally clinically important, as it reflects a person’s inability to live independently. IADL impairment is considered a key element in measuring clinically meaningful treatment effects, because it is related to reduced quality of life, caregiver burden, and apathy [9, 10]. However, a given score on an IADL instrument does not directly indicate whether the level of impairment requires clinical attention [11]. Also, to patients and caregivers, the score itself does not translate to a meaningful concept of problems in daily functioning.

In this study, we set out to investigate the clinical meaningfulness of Amsterdam IADL Questionnaire (A-IADL-Q) scores by establishing clinically meaningful score cutoffs, representing no, mild, moderate and severe problems in daily functioning. Establishing these cutoffs could aid in the meaningful interpretation of A-IADL-Q scores, which could in turn improve communication between clinicians, patients and caregivers.

## Methods

### Participants

We asked informal caregivers of patients who visited our outpatient memory clinic between May and August 2019 to participate in a one-time, 3-h focus group. Additionally, we recruited caregivers through our center’s social media accounts. We approached neurologists, geriatricians, nurse specialists and neuropsychologists from various memory clinics in the Netherlands through contacts of the authors and by using a mailing list for members of the Dutch memory clinics network (Nederlands Geheugenpoli Netwerk).

The study was approved by the ethical review board of the VU University Medical Center, and all participants provided written informed consent.

### Measures

The Amsterdam Instrumental Activities of Daily Living Questionnaire (A-IADL-Q) is an outcome measure that is self-completed by a caregiver and was designed to capture early impairment in daily functioning due to cognitive decline [12]. For the current study, we used the short version of the instrument [13], which consists of a selection of 30 activities from the original 70-item version. Items were selected based on cross-cultural applicability, frequency of endorsement, and clinical relevance, as judged by clinicians, caregivers and patients [13]. Items are rated on a five-point scale ranging from ‘no difficulty performing the activity’ to ‘unable to perform the activity’. The A-IADL-Q is scored using item response theory (IRT), which accounts for varying ‘difficulty’ of items such that impairment in a more complex activity (e.g., managing the household budget) contributes differently to the total score than impairment in a relatively simple activity (e.g., using the TV remote control). This information is contained in the scoring parameters, as described in detail elsewhere [13, 14]. The total score, or *T*-score, represents the latent trait of ‘daily functioning’ and is normally-distributed with a mean of 50 and a standard deviation (SD) of 10 in a memory clinic population. Scores thus range from approximately 20–80, with higher scores representing better daily functioning.

We created nine short clinical summaries (‘vignettes’) of fictional patients who had some degree of functional impairment, using combinations of five items of the A-IADL-Q for each vignette. We selected a subset of fifteen items to reduce the number of different activities presented in each vignette and increase comparability between them. The selection was made based on the IRT parameters to have items distributed across the latent trait, so that both more and less impaired ends of the daily functioning spectrum were covered. We then determined what item response category would be most likely to be endorsed given a certain *T*-score, based on the methods and using an R script adapted from Morgan, Mara [15]. An overview of the most likely item responses of the fifteen items is included in the Additional file 1. The vignettes were created by combining the most likely responses of five items at different *T*-scores (i.e., different degrees of impairment), and were placed five points (0.5 SD units) apart, ranging from 20 (all most likely item responses were ‘unable to perform’) to 60 (all most likely item responses were ‘no difficulty’). We randomly assigned each vignette a gender, common Dutch surname, random age in the range of 60–70 years, and a stock photo. The vignettes can be found in the Additional file 1.

### Procedures

In the focus groups, we asked each panelist to describe what they considered ‘mild’, ‘moderate’ and ‘severe problems’ in daily functioning, to understand how the panelists defined these categories and create a framework for the subsequent categorization and discussion. Subsequently, panelists individually ordered the vignettes from the one representing the least functional impairment to the one representing the most. Panelists then discussed the order of the vignettes and reached a consensus ordering. Then, panelists individually placed bookmarks between the vignettes to create categories representing no, mild, moderate, and severe problems in daily functioning. This ‘bookmarking’ method was previously developed by Cook and colleagues [16]. Finally, a second group discussion resulted in a consensus categorization. Group discussions were based on the nominal group theory [17].

Clinicians individually completed an online survey that was modeled after the focus group procedures, and in which they were first asked to describe what they considered ‘mild’, ‘moderate’ and ‘severe problems’. Next, the nine vignettes were presented in order from least to most impaired, and the clinicians were instructed to categorize them into no, mild, moderate and severe problems.

### Statistical analyses

As the clinicians completed the survey independently, consensus between them was determined by taking the mode of the categorization for each vignette (1 = no problems, 2 = mild problems, 3 = moderate problems, 4 = severe problems). The overall consensus categorization was the mode of the three separate consensus categorizations: two from the focus groups with informal caregivers, and the consensus between clinicians. Analyses were performed in R version 4.1.0 [18].

## Results

Forty patient caregivers were invited through the Alzheimer Center Amsterdam to participate in the focus groups. Six individuals (age 68 ± 10 years old, 4 women) agreed to participate and they were spread across two focus groups. Four panelists were partners, and two were adult children of a person with dementia. Clinicians were approached through contacts of the authors, as well as through a mailing list for clinicians working in memory clinics in The Netherlands. Thirteen clinicians (five neurologists, five nurse specialists, two neuropsychologists and a geriatrician; age 46 ± 13 years old, 8 women) completed the survey.

**Fig. 1:**
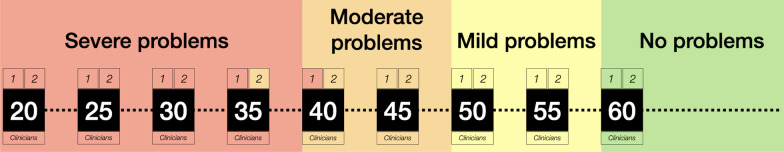
Vignettes and classifications. Each vignette is represented by a black square showing the corresponding T-score. The final classifications as determined in consensus are shown in the background and are color-coded: red for ‘severe problems’, orange for ‘moderate problems’, yellow for ‘mild problems’, and green for ‘no problems’. The consensus classifications per focus group are shown directly above the vignettes (1 = focus group 1, 2 = focus group 2); the consensus classifications for clinicians are shown below

Caregivers and clinicians had differing definitions of what they considered ‘problems in daily functioning’. One caregiver defined ‘problems’ as having any amount of difficulty with performing some activity, whereas another stated that they considered ‘problems’ to be the complete inability to perform an activity. Clinicians wrote that ‘mild problems’ cause minimal impairment predominantly in the most complex activities, whereas ‘severe problems’ imply that a person can no longer function independently. As a result of the various personal definitions, individual categorizations differed slightly, with some panelists categorizing more strictly, where fewer problems were classified as more severe, while others were more lenient, classifying more problems as less severe. Consensus between the focus groups was largely similar, except that in one group, two more vignettes were classified as representing ‘severe problems’, creating a 10-point difference between the cutoffs for ‘severe problems’ in the two groups (see Fig. 1). The vignettes at the extremes, i.e., ‘no problems’ and ‘severe problems’ were classified the same across clinicians and caregivers. The classifications of ‘moderate’ and ‘severe’ problems differed among clinicians, similar to the caregivers.

The final average categorization was as follows: *T*-scores ≥ 60 were classified as showing ‘no problems’, *T*-scores 50–59 were classified as ‘mild problems’, *T*-scores 40–49 as ‘moderate problems’ and *T*-scores < 40 as ‘severe problems’ (Fig. 1).

## Discussion

In this study, we involved stakeholders to determine clinically meaningful scoring categories for the measurement of functional impairment using the Amsterdam IADL Questionnaire. Informal caregivers and clinicians established categories representing no (*T*-score ≥ 60), mild (50–59), moderate (40–49), and severe problems in daily functioning (< 40) in IADL.

Clinical meaningfulness in the context of Alzheimer’s disease and related disorders has been gaining attention over recent years [19, 20]. Clinicians have a good understanding of the disease and its effects on patients and caregivers. Still, when conclusions are based solely on judgments by clinicians, these only comprise part of the picture. Especially, caregivers could add a unique perspective since they observe and can therefore reflect on functioning in AD patients in everyday life. This is a major advantage of our study.

The cutoffs between no and mild, and mild and moderate problems were unanimously agreed upon by caregivers and clinicians. This is especially important, as it seems that clear, clinically meaningful distinctions can be made in subtle degrees of IADL impairment. There was, however, some disagreement among caregivers on the precise placement of a cutoff to make the distinction between moderate and severe problems. It is arguable that the difference between these categories is of less importance, as there is already considerable impairment. Our findings also show that the clinical interpretation may depend on individual definitions and opinions, which has likely contributed to the slight differences we found in categorizations. The categories we present may not reflect everyone’s personal interpretation of different degrees of functional impairment.

Nevertheless, the proposed categories can help clarify the meaning of a given score, and thus provide concrete guidance for communicating test results with patients and their caregivers. This is important as many patients and caregivers report unmet information needs, especially about what test results mean [21, 22]. When discussing test results, communication may benefit from the use of clear language and interpretable categories, rather than raw scores. Our study provides such ready-to-use scoring categories for the Amsterdam IADL Questionnaire.

An important strength of this work is that we used a qualitative approach involving stakeholders (both caregivers and clinicians) to determine clinically meaningful categories in the scoring of a functional outcome measure. Limitations of this study include its small sample size, predominance of women, and recruitment in The Netherlands only, which limit the generalizability of our results. A future study should expand on our work by including a larger sample size representing a more diverse group of caregivers. Future work should also focus on the meaningfulness of changes in daily functioning, as changes may be meaningful, even when they fall entirely within the scoring categories we established here.

## Conclusions

In conclusion, we used caregiver and clinician input to place thresholds and thus create meaningful categories for assessing the severity of impairment in everyday functioning in the context of AD. Specifically, these categories may be useful for distinguishing absence of any problems from the existence of mild problems, which is relevant in early disease stages. Our findings give meaning to total scores, which in and of their own are usually rather unintuitive. By providing clear language about the level of impairment, the categories could support clinicians in explaining the meaning of test results to patients and their caregivers.

## Supplementary Information

**Additional file 1: Additional details on vignettes**. Provides additional information on how the vignettes were made, what activities they are composed of, and lists all vignettes shown to participants.

## Data Availability

The datasets used and/or analyzed during the current study are available from the corresponding author on reasonable request.

## Abbreviations

AD: Alzheimer’s disease
A-IADL-Q: Amsterdam instrumental activities of daily living questionnaire
IADL: Instrumental activities of daily living
SD: Standard deviation
