# Supplementary material for: Giving meaning to the scores of the Amsterdam instrumental activities of daily living questionnaire: a qualitative study
Source: Health Qual Life Outcomes. 2022 Mar 24;20:47. doi: 10.1186/s12955-022-01958-2 (PMC8943938; doi:10.1186/s12955-022-01958-2)
Supplement: Supplementary file 1 — Additional file 1: Additional details on vignettes. Provides additional information on how the vignettes were made, what activities they are composed of, and lists all vignettes shown to participants. [file 12955_2022_1958_MOESM1_ESM.docx]

# Additional file 1

# Appendix A – Vignette creation

We adapted an R script from Morgan and colleagues^14^ to obtain the most likely responses from the IRT scoring parameters, and selected fifteen optimal items to be included in the vignettes based on: (a) the distribution across the latent trait, so as to include both relatively easy and relatively difficult activities, and (b) endorsement levels, to ensure that all activities described in the vignettes were widely relevant.

The table below shows the response categories most likely to be selected at each *T*-score.

Table 1. Most likely response categories for the items used in the vignettes.

| **Item content** | **T=20** | **T=25** | **T=30** | **T=35** | **T=40** | **T=45** | **T=50** | **T=55** | **T=60** |
| --- | --- | --- | --- | --- | --- | --- | --- | --- | --- |
| Cooking | UN | UN | UN | UN | MD | MD | SMD | SMD | ND |
| Preparing sandwiches | UN | UN | MD | SMD | SMD | ND | ND | ND | ND |
| Using household appliances | UN | UN | MMD | MD | MD | SMD | SMD | ND | ND |
| Using the microwave | UN | UN | UN | MMD | MD | SMD | ND | ND | ND |
| Paying bills | UN | UN | UN | UN | UN | MD | SMD | ND | ND |
| Managing the paperwork | UN | UN | UN | UN | UN | MMD | SMD | SMD | ND |
| Withdrawing cash from an ATM | UN | UN | UN | UN | MD | SMD | ND | ND | ND |
| Paying with cash | UN | UN | MMD | MD | SMD | SMD | ND | ND | ND |
| Making and keeping appointments | UN | UN | UN | UN | MMD | MD | SMD | SMD | ND |
| Filling in forms | UN | UN | UN | UN | MMD | MD | SMD | SMD | ND |
| Working | UN | UN | UN | UN | UN | MMD | MD | SMD | ND |
| Using the TV remote control | UN | MMD | MMD | MD | SMD | SMD | ND | ND | ND |
| Driving a car | UN | UN | UN | UN | MD | SMD | SMD | ND | ND |
| Using public transportation | UN | UN | UN | UN | MMD | MD | SMD | ND | ND |
| Being responsible for their medication | UN | UN | MMD | MD | SMD | SMD | SMD | ND | ND |

Higher *T*-scores represent better overall IADL functioning. Item responses are coded as follows: UN, unable to perform the activity; MMD, much more difficulty; MD, more difficulty; SMD, somewhat more difficulty; ND, no difficulty.

# Appendix B – Focus group clinical summaries (translated from Dutch)

## 1. Ms. Jonker (65 years old) [*T* = 60]

In the past four weeks, Ms. Jonker did not have any difficulty cooking. She also did not experience any difficulty using household appliances. She had no difficulty making and keeping appointments. She also did not have any difficulty using the TV remote control. She did not have any difficulty being responsible for her own medication.

In summary, Ms. Jonker:

- did not have any difficulty cooking,
- did not have any difficulty using household appliances,
- did not have any difficulty making and keeping appointments,
- did not have any difficulty using the TV remote control, and
- did not have any difficulty being responsible for her own medication.

## 2. Ms. Smit (63 years old) [*T* = 55]

Ms. Smit did not have any difficulty preparing sandwiches in the past four weeks. She also did not have any difficulty obtaining the correct amount of cash from an ATM. She did not have any difficulty using public transportation. She did have somewhat more difficulty managing the paperwork than she had in the past. She also did find it somewhat more difficult to fill in forms than she had in the past.

In summary, Ms. Smit:

- did not have any difficulty preparing sandwiches,
- did not have any difficulty obtaining the correct amount of cash from an ATM,
- did not have any difficulty using public transportation, but
- had somewhat more difficulty managing the paperwork than she had in the past, and
- had somewhat more difficulty filling out forms than she had in the past.

## 3. Ms. Prins (68 years old) [*T* = 50]

Ms. Prins did not have any difficulty paying with cash in the past four weeks. She did have somewhat more difficulty cooking, as well as somewhat more difficulty using household appliances, than she had in the past. She also had somewhat more difficulty paying the bills than she had in the past. She had more difficulty working than she had in the past.

In summary, Ms. Prins:

- did not have any difficulty paying with cash, but
- had somewhat more difficulty cooking than she had in the past,
- had somewhat more difficulty using household appliances than she had in the past,
- had somewhat more difficulty paying the bills than she had in the past, and
- had more difficulty working than she had in the past.

## 4. Mr. Molenaar (62 years old) [*T* = 45]

In the past four weeks, Mr. Molenaar had no difficulty using the TV remote control. However, he did have somewhat more difficulty using the microwave than he had in the past. He also had somewhat more difficulty driving a car than he had in the past. He had more difficulty making and keeping appointments. He had much more difficulty managing the paperwork than he had in the past.

In summary, Mr. Molenaar:

- did not have any difficulty using the TV remote control, but
- had somewhat more difficulty using the microwave than he had in the past,
- had somewhat more difficulty driving a car than he had in the past,
- had more difficulty making and keeping appointments than he had in the past, and
- had much more difficulty managing the paperwork than he had in the past.

## 5. Mr. Blom (61 years old) [*T* = 40]

In the past four weeks, Mr. Blom had somewhat more difficulty preparing sandwiches than he had in the past. He also had somewhat more difficulty being responsible for his own medication than he had in the past. He had more difficulty cooking, and also had more difficulty using public transportation than he had in the past. He was no longer able to work.

In summary, Mr. Blom:

- had somewhat more difficulty preparing sandwiches than he had in the past,
- had somewhat more difficulty being responsible for his medication than he had in the past,
- had more difficulty cooking than he had in the past,
- had much more difficulty using public transportation than he had in the past, and
- was no longer able to work.

## 6. Mr. Dekker (63 years old) [*T* = 35]

Mr. Dekker had more difficulty using household appliances in the past four weeks than he had in the past. He also had more difficulty paying with cash than he had in the past. He also had more difficulty using the TV remote control than he had in the past. He had much more difficulty using the microwave than he had in the past. He was no longer able to make and keep appointments.

In summary, Mr. Dekker:

- had more difficulty using household appliances than he had in the past,
- had more difficulty paying with cash than he had in the past,
- had more difficulty using the TV remote control than he had in the past,
- had much more difficulty using the microwave than he had in the past, and
- he was no longer able to make and keep appointments.

## 7. Ms. Vermeulen (60 years old) [*T* = 30]

In the past four weeks, Ms. Vermeulen had more difficulty preparing sandwiches than she had in the past. She had much more difficulty being responsible for her own medication than she had in the past. She was no longer able to pay the bills. She was also no longer able to manage the paperwork, and was no longer able to work.

In summary, Ms. Vermeulen:

- had more difficulty preparing sandwiches than she had in the past,
- had much more difficulty being responsible for her own medication than she had in the past,
- was no longer able to pay the bills,
- was no longer able to manage the paperwork, and
- was no longer able to work.

## 8. Mr. De Vries (66 years old) [*T* = 25]

In the past four weeks, Mr. De Vries had much more difficulty using the TV remote control than he had in the past. He was no longer able to use the microwave. He was also no longer able to make or keep appointments. He was no longer able to fill in forms. He was also no longer able to use public transportation.

In summary, Mr. De Vries:

- had much more difficulty using the TV remote control than he had in the past,
- was no longer able to use the microwave,
- was no longer able to make or keep appointments,
- was no longer able to fill in forms, and
- was no longer able to use public transportation.

## 9. Ms. De Ruiter (66 years old) [*T* = 20]

In the past four weeks, Ms. De Ruiter was no longer able to prepare sandwiches. She was also no longer able to use household appliances, or to manage the paperwork. She was no longer able to obtain the correct amount of cash from an ATM. She was no longer able to drive a car.

In summary, Ms. De Ruiter:

- was no longer able to prepare sandwiches,
- was no longer able to use household appliances,
- was no longer able to manage the paperwork,
- was no longer able to obtain the correct amount of cash from an ATM, and
- was no longer able to drive a car.
